# Assessment of the clinical and cost-effectiveness evidence in the reimbursement decisions of new cancer drugs

**DOI:** 10.1016/j.esmoop.2022.100569

**Published:** 2022-08-28

**Authors:** G. Chauca Strand, C. Bonander, N. Jakobsson, N. Johansson, M. Svensson

**Affiliations:** 1 Health Economics and Policy, School of Public Health and Community Medicine, Institute of Medicine, University of Gothenburg, Gothenburg; 2 Karlstad Business School, Karlstad University Faculty of Arts and Social Sciences, Karlstads Business School, Karlstad; 3 University Health Care Research Center, Faculty of Medicine and Health, Örebro University, Örebro, Sweden; 4 Department of Pharmaceutical Outcomes and Policy, University of Florida, Gainesville, Florida, USA

**Keywords:** reimbursement, health technology assessment, decision making, cancer drugs

## Abstract

**Background:**

This study aimed to describe the clinical and cost-effectiveness evidence supporting reimbursement decisions of new cancer drugs and analyze the influence of trial characteristics and the cost per quality-adjusted life years (QALYs) on the likelihood of reimbursement in Sweden.

**Patients and methods:**

Data were extracted from all appraisal dossiers for new cancer drugs seeking reimbursement in Sweden and claiming added therapeutical value between the years 2010 and 2020. The data were analyzed using descriptive statistics, and logistic regression models were also used with the cost per QALY, study design, comparator, and evidence on final outcomes in the clinical trials as predictors of reimbursement.

**Results:**

All 60 included appraisals were based on trial evidence that assessed at least one final outcome (overall survival [OS] or quality of life [QoL]), although rarely as a primary outcome. Of the appraisals with a final decision (*n* = 58), 79% were approved for reimbursement. Among the reimbursed drugs, only half had trial evidence demonstrating improved OS or QoL. Only one drug had trial evidence supporting improvements in both OS and QoL. The average cost per QALY for reimbursed cancer drugs was estimated to be 748 560 SEK (€73 583). A higher cost per QALY was found to decrease the likelihood of reimbursement by 9.4% for every 100 000 SEK (€9830) higher cost per QALY (*P* = 0.03). For cost-effectiveness models without direct evidence of improvements in final outcomes, a larger QALY gain was observed compared with those with evidence mainly relying on intermediate and surrogate outcomes.

**Conclusions:**

There are substantial uncertainties in the clinical and cost-effectiveness evidence underlying reimbursement decisions of new cancer drugs. Decision makers should be cautious of the limited evidence on patient-centered outcomes and the implications of allocating resources to expensive treatments with uncertain value for money.

## Highlights

• Most cancer drug indications lacked evidence on the effects on OS or QoL at reimbursement decision.
• The mean cost per QALY for reimbursed cancer drugs was estimated to 750 000 SEK (€73 600).
• In cost-effectiveness models, QALY gains were larger when input came from trials relying on intermediate outcomes.
• The clinical and economic evidence used for reimbursement decisions is characterized by uncertainties and risk of bias.

## Introduction

Many countries have experienced a rise in health care expenditures due to cancer over the last decade.^1^ This trend has been explained by increasing cancer incidence and higher per-patient treatment costs following an increasing use of expensive pharmaceutical treatments.^1^^,^^2^ In recent years, new cancer drugs have significantly increased in numbers^3^, ^4^, ^5^ and price^3^^,^^6^^,^^7^ in Europe and the United States. Although advances in cancer medication have generated benefits over the years, the increase in costs has raised questions regarding the affordability and sustainability of funding many new drugs within health care systems^8^^,^^9^ and, increasingly, their value for money.

While many countries accept high costs for drugs with substantial beneficial effects on mortality and morbidity, there is a parallel discussion on the evidence of the clinical value of new cancer drugs. Previous research has shown that an essential share of market authorizations of new cancer drugs in Europe and the United States has low to intermediate benefit^10^ and has been based on limited evidence on quality of life (QoL)^11^ and overall survival (OS) at the time for approval, with a large share of new drugs relying on evidence on intermediate and surrogate outcomes.^12^, ^13^, ^14^ The limited, and in many cases, lack of, evidence on patient-centered outcomes is problematic when considering the needs and preferences of the targeted population. Furthermore, the extensive use of surrogate measures for patient-centered outcomes has been questioned.^15^^,^^16^ While certain studies have validated surrogates for specific cancer therapies and indications,^17^, ^18^, ^19^, ^20^ many of the commonly used surrogate measures in cancer have shown weak or lack of validation for OS.^13^^,^^16^^,^^21^, ^22^, ^23^, ^24^

As limited resources are increasingly challenging health care systems with continued growth in health care costs, the use of health economic evaluations to acquire valuable information about efficient resource allocations has grown. Economic evaluations are reportedly being considered across many different reimbursement systems^25^ and it has been shown that cost-effectiveness results influence reimbursement decisions in many countries.^26^, ^27^, ^28^, ^29^, ^30^ However, as drugs are approved with limited evidence of their clinical efficacy and relative effectiveness, the reliability and usefulness of the economic evaluations may be challenged.

Previous studies have assessed possible influential factors on decision making and found, for example, the reported clinical benefit, level of severity, orphan designation status, study design, treatment intent, and the availability of alternative treatments to influence reimbursement decisions for cancer drugs and drugs in general.^26^^,^^28^^,^^31^, ^32^, ^33^, ^34^ Concerns regarding the quality of evidence and uncertainty when assessing clinical benefits and effectiveness used in cost-effectiveness analyses and reimbursement decisions have been emphasized.^35^, ^36^, ^37^ Studies on the available evidence for cancer drugs have primarily assessed the evidence used for market authorizations and found that evidence for the clinical benefit is limited regarding the effects on patient-centered outcomes such as survival and QoL.^11^^,^^12^^,^^14^^,^^38^ In a recent study, the clinical benefit of cancer drugs evaluated for reimbursement recommendations also showed that just around half of the drugs recommended for reimbursement demonstrated improvements on OS.^39^ However, few studies have explored both the clinical and economic evidence used in the decision-making process for the reimbursement of cancer drugs and their relative importance. This paper aimed to describe and analyze the clinical and cost-effectiveness evidence used to support new cancer drugs’ reimbursement decisions between 2010 and 2020 in Sweden, focusing specifically on the availability of patient-centered outcomes.

## Materials and methods

### Institutional context

In Sweden, reimbursement decisions for prescription drugs are primarily based on clinical effectiveness, cost-effectiveness, need, and severity.^40^^,^^41^ The process is initiated by pharmaceutical companies who submit a reimbursement application to the Swedish Dental and Pharmaceutical Benefits Agency (TLV). The producer provides the underlying clinical evidence, proposes the price, and provides a cost-effectiveness analysis based on the suggested price. The reimbursement unit at TLV assesses the applications and, if deemed necessary, makes adjustments to modeling assumptions for the economic evaluations. The final decision, as well as a severity statement of the targeted disease, is made by the Board of Pharmaceutical Benefits, constituted by independent professionals from different sectors of academia, health regions, and patient organizations. Decisions can lead to full, restricted, conditional, or rejected reimbursement. A full approval implies that a drug is reimbursed for all of its indications, and a restricted approval indicates that reimbursement is given for a specific indication or a subgroup of patients. For a conditional approval, either a full or restricted reimbursement can be granted given that certain conditions are fulfilled, such as requirements for the producer to provide follow-up data on, for example, the usage of the drug in real clinical practice.

### Sample selection

TLV compiled a list with a total of 127 applications concerning cancer drugs upon the authors’ request of all appraisals regarding new cancer drugs between 2010 and 2020. The appraisal dossiers and decisions made by TLV are regarded as public documents and are published on their website. The list served as a basis for the sample selection and was reviewed against the official database. All applications classified as cancer drugs by the agency with available appraisal dossiers were retrieved from the official database or directly handed by the agency. The dossiers were included if the application concerned a new pharmaceutical targeting a cancer indication with claimed added therapeutical value or had an economic evaluation constituted by a cost-effectiveness analysis. A new pharmaceutical was defined as ‘Originator drugs with active substances that are not already reimbursed, pharmaceuticals with already reimbursed active substances having a new indication, generic drugs where no pharmaceutical (originator or generic) have the same active substance reimbursed as well as biosimilars.’^41^ In addition, pharmaceuticals with new pharmaceutical strength or dosage form targeting a new patient group or indication were considered. Applications including a price comparison or cost-minimization analysis (*n* = 32) as the main economic evaluation were excluded because most concerned generics. Withdrawals with missing documents (*n* = 16), duplicates, and reassessments of follow-ups were likewise excluded due to the scope of the study. The selection resulted in 56 dossiers comprising 60 appraisals eligible for analysis. For an illustration of the selection and list of drugs included, see Supplementary Figure S1 and Table S1, available at https://doi.org/10.1016/j.esmoop.2022.100569.

### Data extraction

Data were extracted from the official dossiers and decision summaries from TLV. The extraction was based on the number of appraisals for specific drug indications included in the dossiers rather than the number of drugs, as the same drug could apply for reimbursement for several indications. The included appraisals were extracted based on a prespecified data template focusing on the clinical and cost-effectiveness evidence explicitly reported in the submissions (see Supplementary Table S2, available at https://doi.org/10.1016/j.esmoop.2022.100569).

### Assessment of clinical evidence

Data for the clinical evidence were extracted on the type of endpoints used, the type of study design, the choice of comparator, and whether OS or QoL outcomes was included in the clinical trial. The endpoints included in a trial were assessed either as a ‘final outcome’ or as an intermediate/surrogate outcome. A final outcome was defined when a trial assessed the effects on OS or QoL (or both), while an intermediate outcome was defined as any other measure assessed (e.g. progression-free survival, time-to-progression, response rate). Further, data on whether a statistically significant result was found on a final outcome (*P* value ≤ 0.05), the estimated change in QoL, median OS, and progression-free survival gain in months were extracted.

### Assessment of cost-effectiveness evidence

Data on the cost-effectiveness evidence were extracted based on the choice of comparator (e.g. current treatment, no treatment), how the comparative effects were established (e.g. randomized controlled trial, single arm, indirect comparison), and results on the incremental cost-effectiveness ratio measured as the cost per quality-adjusted life year (QALY). Information on dominating and dominated results in the cost-effectiveness analyses was extracted but the observations were excluded in further analyses (*n* = 2). Additional data were extracted, including the QALY gain, type of application, the number of eligible patients, and the final decision. In some cases, the estimated cost per QALY was reported as an interval. In such cases, the average and the lower and upper bounds of these intervals were analyzed. The average value was used in the main analyses and is referred to in the results.

### Statistical analysis

Descriptive statistics were used to analyze the type of clinical and cost-effectiveness evidence. Factors assessed included the type of primary endpoint used, type of comparator, study design, and the type of treatment alternative used in the economic evaluation. Likewise, the cost per QALY and the type of comparison made to establish the comparative effects in the cost-effectiveness analyses were analyzed. To assess the association and influence of the different evidence types on the final reimbursement decision and enable further analyses, the variables were categorized into binary variables (Table 1). Because of the small number of observations, the final decision was categorized as an ‘Approval’ or ‘Rejection’, for which decisions for full, restricted, and conditional reimbursement were regarded as approval.

**Table 1:** Variables used in the statistical analyses

	N	Description	Value
Cost per QALY	53	The incremental cost-effectiveness ratio	Continuous
Study design	60	Study design of the clinical trial	0 if non-RCT 1 if RCT
Primary endpoint	60	The type of endpoint used as primary endpoint in the clinical trial	0 if intermediate outcome 1 if final outcome
Direct evidence on final outcomes	58	Whether the evidence on clinical benefits were based on demonstrated and reported statistically significant results on final outcomes (OS *or* QoL) or intermediate outcomes	0 if no 1 if yes
Level of severity	58	Classification made by the agency of the level of severity of the targeted disease^a^	0 if high 1 if very high
Treatment alternative	60	Treatment alternative used as comparator in the economic evaluation.	0 if active comparator 1 if no comparator
Type of comparison	60	Type of comparison made to assess the comparative effectiveness of treatment alternatives in the CEA	0 if indirect comparison 1 if direct comparison

CEA, cost-effectiveness analysis; OS, overall survival; QALY, quality-adjusted life year; QoL, quality of life; RCT; randomized controlled trial.

a One observation was classified as ranging between medium-high level of severity and was categorized as having a ‘high’ level of severity. No disease was classified as having low severity.

To assess whether direct evidence on a final outcome was used as an evidence basis in the decision process, a trial was defined to have direct evidence of improvements in final outcomes if having statistically significant results on a final outcome irrespective of assessed as a primary or secondary endpoint. Appraisals that did not report results on final outcomes assessed in the trial or did not include statistical information of the results were defined as not having stated a statistically significant result (Table 1).

Logistic regression analyses were performed to estimate the influence of the variables on the reimbursement decision. Several models were created to include the different variables as predictors while also considering the small sample size. All models had the reimbursement decision as an outcome. The predictors consisted of the cost per QALY, study design, type of evidence, type of comparison made in the economic evaluation, and treatment alternative in different combinations (see Supplementary Table S3, available at https://doi.org/10.1016/j.esmoop.2022.100569). Because of the lack of variation in the level of severity statement (all having the highest level of severity in the classification scales over the time period), this variable was not included in the model specifications. The models were compared and selected based on the results from the Akaike information criterion primarily and Bayesian information criterion tests for model fit and selection. All analyses were performed using Stata 16 (StataCorp LLC, TX).

### Ethics

The study did not involve individual or patient data. No ethical permission was therefore required.

## Results

A total of 60 appraisals were included for analysis. The number of annual decisions made over time varied, with the highest number in 2015 (20%). Overall, approval was granted for 79% of all appraisals with a final decision. For two of the appraisals, a final decision was not made due to a late withdrawal from the pharmaceutical company.

### Clinical evidence used in applications

Table 2 displays the characteristics of the trials used to assess the clinical and economic evidence. For all 60 appraisals, 82% of the evidence was supported by at least one randomized controlled trial, while the remaining were supported by single-arm studies. All trials included and assessed OS; however, intermediate outcomes measures (such as progression-free survival and objective response rate) were used as the primary endpoint in most evaluated trials (80%).

**Table 2:** Description of the cost per QALY and characteristics of trials composing the clinical evidence

	Overall	Approvals	Rejected
	*n* (%)	*n* (%)	*n* (%)
Study design:			
Double-blinded RCT	29 (48)	24 (52)	4 (33)
Open RCT	20 (33)	15 (33)	4 (33)
Single-arm trial	11 (18)	7 (15)	4 (33)
Comparator:			
Active control	23 (38)	18 (39)	4 (33)
Placebo	26 (43)	21 (46)	4 (33)
No control	11 (18)	7 (15)	4 (33)
Primary outcome:			
Final outcome	12 (20)	8 (17)	4 (33)
Intermediate outcome	48 (80)	38 (83)	8 (67)
Results on OS:			
Yes	25 (42)	19 (41)	5 (42)
No	15 (24)	12 (26)	2 (17)
Not stated	18 (31)	14 (30)	4 (33)
Confidential	2 (3)	1 (2)	1 (8)
Results on QoL:			
Yes	6 (10) 7 (12) 18 (30)	4 (9)	1 (8)
No		5 (11)	2 (17)
Not stated		16 (35)	2 (17)
Not applicable	29 (48)	21 (46)	7 (58)
Cost per QALY^a^			
Mean	749 000	1 384 000	
Median	785 000	1 130 000	
IQR	240 000	550 000	
SD	213 000	508 000	

Not stated indicates that no statistical information or result was provided including observations with immature data; IQR, interquartile range; OS, overall survival; QALY, quality adjusted life years; QoL, quality of life; RCT, randomized controlled trial; SD, standard deviation.

a Cost per QALY in Swedish krona, 1 SEK = €0.0983.

Regarding the evidence on OS and QoL, 42% of the appraisals relied on trials that provided a statistically significant result on OS, while the remaining did not report a statistically significant result or provide any statistical information on the results (Table 2). Few trials reported complete evidence on the effects on QoL. While 52% of the trials reportedly assessed QoL outcomes, most did not report any results or provide any statistical information regarding the result (58%). In total, a statistically significant improvement in QoL could only be established in six of the evaluated trials (10%).

### Clinical evidence for the reimbursed drugs

**Figure 1:**
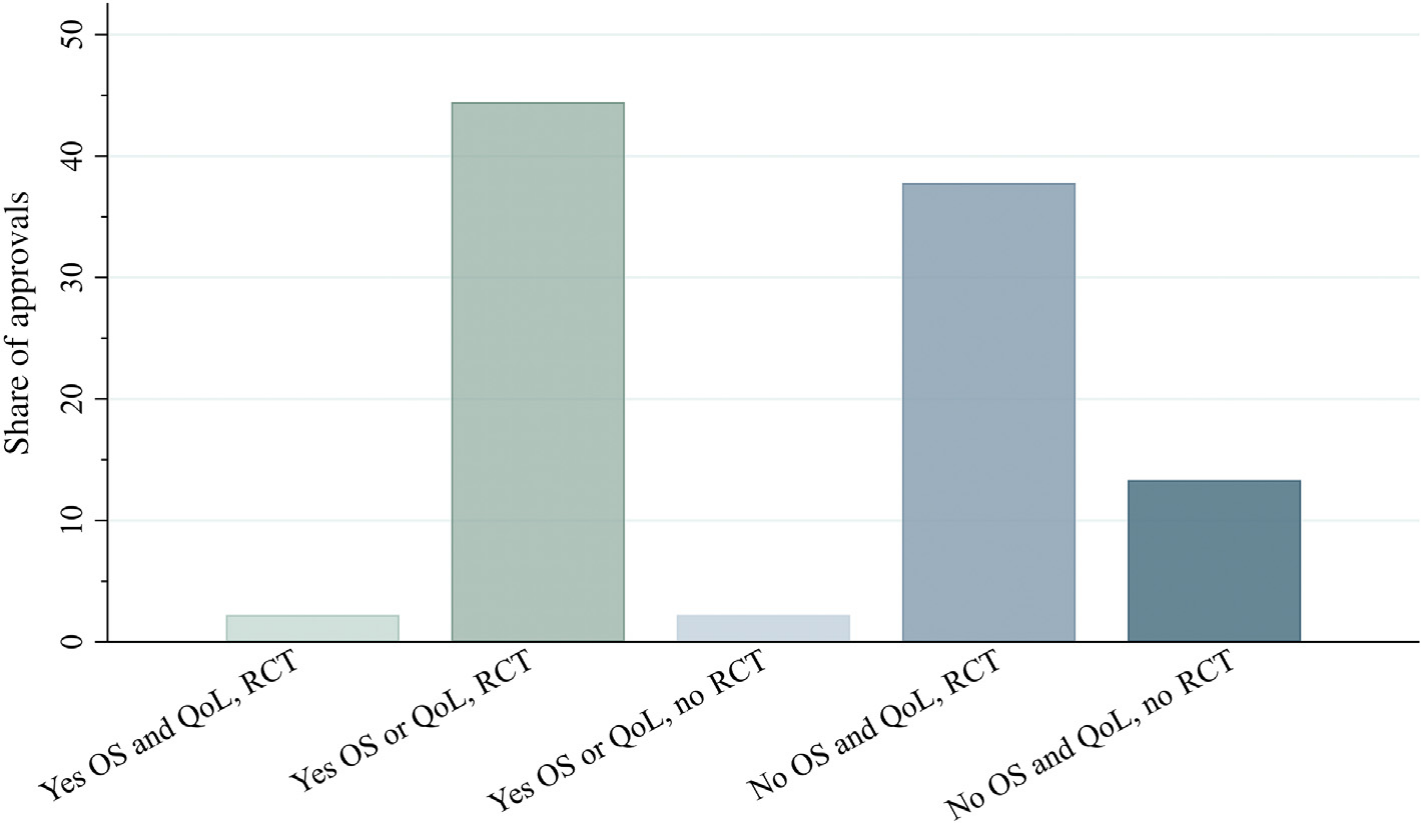
Characteristics of trials constituting evidence for reimbursed drug indications. Share of the number of approvals (*n* = 45) is presented in percent. Observations with confidential results on OS were excluded (*n* = 1). No indicates that no statistically significant result was reported on the outcome and includes observations with nonstatistically significant results or with no statistical information or result provided. Yes indicates that a statistically significant result was provided on the outcome. OS, overall survival; QoL, quality of life; RCT, randomized controlled trial.

The combined characteristics of the clinical evidence used for reimbursed drug indications can be seen in Figure 1. For most approvals, there was limited evidence on final outcomes. In 53% of the approvals with statistically significant improvements on OS, irrespective of study design, effects on QoL had not been assessed. A lack of statistical information and reported results on QoL were also seen for most trials without evidence on OS. In half of the approvals, evidence was based on trials without statistically significant results provided for OS or QoL, and ∼26% of these (13% of total) were based on single-arm studies. Only one reimbursed drug indication had evidence based on trials that demonstrated a statistically significant improvement on both OS and QoL at the time of approval.

### Cost-effectiveness evidence

The trials upon which the clinical evidence was based were all used for the economic evaluation. However, when further analyzing the economic evidence used to support the decision of reimbursement, it was found that around one-third (35%) of all the economic evaluations in the applications relied on indirect comparisons to establish the comparative effectiveness of the treatment alternatives. Hence, in these cases, the comparator in the trial was not considered as the most appropriate for the reimbursement decision.

**Figure 2:**
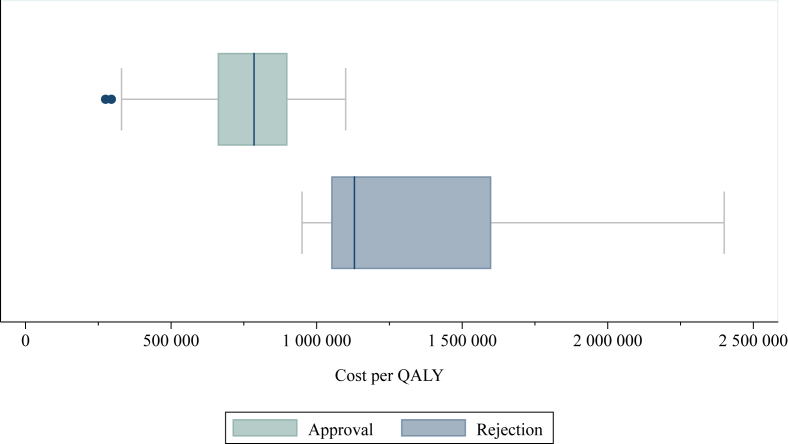
**Boxplot of the cost per QALY over accepted and rejected reimbursements**. Cost per QALY in Swedish krona (1 SEK = €0.0983). For two appraisals that were excluded in this graph, the evaluated drug was found to be dominating or dominated. The dominated drug was rejected for reimbursement. QALY, quality-adjusted life year.

The mean cost per QALY for approved and rejected reimbursements was estimated to 748 560 SEK (€73 583) and 1 384 200 SEK (€136 067), respectively (Swedish krona converted to euros using the European Central Bank’s exchange rate, 1 SEK = €0.0983). The cost per QALY was found to overlap and ranged between 275 000 and 1 100 000 SEK (€27 033 and 108 130) and 950 000 and 2 400 000 SEK (€93 385 and 235 920) for approved and rejected reimbursements, respectively (Figure 2). The cost per QALY varied between the different approvals, with the restricted and conditional approvals having the highest incremental cost-effectiveness ratios. The differences were statistically significant, and an association was found between the decision outcome and cost per QALY (see Supplementary Table S4, available at https://doi.org/10.1016/j.esmoop.2022.100569). In addition, a statistically significant difference was found in the estimated QALY gain in the economic evaluations, where observations with direct evidence on final outcomes had smaller QALY gains compared with those without (see Supplementary Table S5, available at https://doi.org/10.1016/j.esmoop.2022.100569).

### Factors influencing reimbursement decisions

Table 3 shows the logistic regression results for the models with the best statistical fit and coefficients presented as marginal effects (difference in the probability of reimbursement). The cost per QALY is displayed in terms of 100 000 SEK increases. In model 1, the cost per QALY can be interpreted as an increase of 100 000 SEK being associated with a 9.5% lower probability of reimbursement. Having a trial with direct evidence on final outcomes (i.e. statistically significant evidence on OS or QoL) was associated with an 8.2% lower likelihood of reimbursement compared with only having evidence on an intermediate outcome, although the result was not statistically significant.

**Table 3:** Logistic regressions on the likelihood of reimbursement: all models

	(1)	(2)	(3)	(4)	(5)
Cost per QALY	−0.095^a^ (0.015)	−0.094^b^ (0.025)	−0.107^b^ (0.018)	−0.094^b^ (0.025)	−0.098^b^ (0.032)
Direct evidence on final outcomes: Yes		−0.082 (0.058)		−0.082 (0.062)	−0.065 (0.065)
RCT			−0.135 (0.084)		
No treatment alternative				0.000 (0.059)	
Direct comparison					−0.034 (0.068)
N	53	51	53	51	51
Pseudo R^2^	0.658	0.741	0.713	0.740	0.746
Akaike information criterion		17.49	19.84	19.49	19.23

The cost per QALY is displayed in terms of 100 000 SEK increases.

Results in marginal effects. Standard errors are in parenthesis.

QALY, quality-adjusted life years; RCT, randomized controlled trial.

a *P* < 0.01.

b *P* < 0.05.

The results for the remaining predictors are presented in Table 3 and were likewise not found to be statistically significant and only slightly changed the magnitude from model 1 on the association of the cost per QALY. For results on the full model specification, see Supplementary Table S6, available at https://doi.org/10.1016/j.esmoop.2022.100569.

## Discussion

This study investigated the use and influence of clinical and economic evidence for reimbursement decisions of new cancer drugs in Sweden. The results indicate that a large share of the clinical and cost-effectiveness evidence is surrounded by large uncertainties. In most of the clinical trials examined, the effects on QoL were not provided, and less than half showed statistically significant results on OS. Extensive use of intermediate outcome measures as primary endpoints was observed and in a considerable share of cost-effectiveness analyses, indirect comparisons were made to establish the comparative effectiveness of treatments.

The findings on the extensive use of intermediate, surrogate outcomes and limited evidence on OS and QoL align with previous studies on the available evidence for authorized cancer drugs and reimbursement recommendations.^11^, ^12^, ^13^^,^^38^^,^^39^ Compared with previous research evaluating the evidence of both OS and QoL for cancer drugs within Europe, this study found a slightly higher share of drugs based on single-arm studies, and without direct evidence on OS and QoL.^12^ While the greater use of single-arm studies likely is a result of increasing developments for orphan designations in oncology,^42^ it generates greater uncertainty of the evidence on clinical benefit. As we are using newer data than previous studies, our results may indicate that these limitations increase over time.

The effects on OS and QoL were supported by limited evidence and were in many cases not provided, which is problematic regarding the needs and preferences of the targeted population. It has been emphasized that patients expect larger benefits for the hardships of treatments than current evidence may suggest^43^^,^^44^ and that many patients with very short life expectancy prefer improved QoL through, for instance, relief of pain and discomfort.^44^ While having OS as a secondary endpoint in a trial could require a longer time to demonstrate evidence, and benefits for OS may be found after longer follow-up and increased data maturity for some drugs, uncertainty remains about how often this may be the case. In a recent study, Cherny^10^ observed a lack of evidence of OS benefit also after a shorter follow-up from approval in the advanced setting and found that a significant share of approved drugs proved a low or intermediate magnitude of benefit using the ESMO-MCBS (ESMO-Magnitude of Clinical Benefit Scale) scale.

The reliance on intermediate, surrogate outcomes to inform and predict OS and QoL clinical effectiveness is also a source of uncertainty. While there are examples of valid intermediate outcomes, surrogates for specific cancer indications and therapies,^17^, ^18^, ^19^, ^20^ studies have also emphasized a lack of validation for endpoints used as surrogates for OS and QoL for various cancer indications.^15^^,^^16^^,^^22^, ^23^, ^24^^,^^45^ Several drugs with approval based on intermediate, surrogate endpoints have been shown to lack evidence on the effects on OS after later follow-up,^12^, ^13^, ^14^ and some have even been found to have adverse side effects without having any significant effect on OS.^46^ Furthermore, the lack of direct evidence on OS and QoL has negative implications for the validity of cost-effectiveness analyses and decisions regarding the optimal use of resources within health care systems.

The cost per QALY was negatively associated with the likelihood of reimbursement, which demonstrates the importance of cost-effectiveness in the decision-making process. This is in line with the operationalization of the cost-effectiveness principle in Sweden and with previous studies on decision making within health technology assessment authorities.^26^^,^^27^^,^^29^^,^^47^ However, the analysis of the influence of having direct evidence on final outcomes showed a negative relationship between the probability of reimbursement and observations with direct evidence of improvements on OS or QoL compared with those without. This was seen in all model specifications, but was not statistically significant. The result is similar to the findings of Pinto et al.,^30^ whose results indicated that drugs with available OS benefits were less likely to be recommended by National Institute for Health and Care Excellence (NICE). The lack of influence of direct evidence of improvements on both OS and QoL raises questions about how different outcomes are valued as a clinical benefit for cancer diseases. While there may be valid reasons for the lack of evidence on OS, such as for indications in curative settings with long survival expectancy, or cases of rare disease or severity that could reduce the influence of having evidence on OS, more than half of the observations in this analysis had trials used as clinical evidence without direct evidence on neither OS nor QoL. Furthermore, we found that the average QALY gain in the economic evaluations was significantly lower for those with direct evidence on OS or QoL as compared with those having only evidence on intermediate, surrogate outcomes. In the absence of direct evidence on final outcomes, economic evaluation requires extrapolation on immature data, indirect comparisons, or assumptions of the relationship between intermediate outcomes, QoL, and OS. The overall findings call into question how evidence on intermediate outcomes is regarded when evidence on final outcomes is limited and uncertain. As many intermediate outcomes used as surrogates remain debated and unvalidated, but increasingly are being used,^15^ the results observed here raise potential questions of whether optimistic assumptions and overestimations of the added benefits of these drugs currently are being generated, through the use of evidence on intermediate outcomes.

As evidence of clinical effectiveness directly impacts the results of economic evaluations, it is crucial to reflect upon how these uncertainties may bias cost-effectiveness results and information given to decision makers, especially as the estimated mean cost per QALY for cancer drugs observed was high compared with other drugs targeting severe diseases in Sweden.^48^ The limited and uncertain evidence on final outcomes and large use of indirect comparisons in economic evaluations along with a high accepted cost per QALY is a problematic mix.

Additional concerns with the clinical and economic evidence supporting reimbursement of new cancer drugs not directly analyzed in this study include the use of suboptimal controls,^49^^,^^50^ which has been reported for ∼25% of Food and Drug Administration (FDA)-approved cancer drugs between the years 2014 and 2019,^50^ the prevalence of unrepresentative trial samples,^51^ and cross-over.^50^ As resources are scarce within health care systems, reimbursement decisions based on low-quality evidence can adversely affect cancer patients in need of efficient medications as well as patients in other disease areas that see displaced care when implementing new expensive treatments with unclear benefits. Further research is needed regarding the validation and use of intermediate, surrogate outcomes; the external validity of clinical trials used as evidence; as well as a follow-up and monitoring of currently accepted drugs to ensure meaningful benefits within health care systems.

### Limitations

The main limitations of the present study concern the small sample size, especially the small number of rejections. The models also depend on simplifying assumptions that may affect the estimated associations. For instance, conditional and unconditional approvals were analyzed as one category due to the small sample size, even though they may convey different levels of skepticism toward the underlying evidence. Furthermore, due to the complexity of the disease condition and decision-making process, the models included may miss factors that are of importance for the reimbursement decision such as orphan designation, treatment setting, which are not controlled for here*.* Data extraction was made by one reviewer which possibly may limit the objectivity of the underlying information. However, this was minimized as far as possible by the use of a data-extraction template to guide the extraction. Because of the limited scope of the study and the primary aim of assessing the results on OS and QoL, no data on the statistical significance for observations being assessed as intermediate outcomes were extracted. Thus some trials being assessed as surrogate based may be without significant results on the endpoint in question. However, if that is the case, this would mean that the study underestimates the uncertainties in the evidence used to support reimbursement decisions. This study further focused on Sweden, and it is likely that differences exist between different reimbursement systems. However, an analysis of the introduction, and availability of cancer drugs through reimbursement in Europe, showed a similar rate of availability in Sweden and other European countries such as The Netherlands, France, and England for oncology drugs approved in 2017-2022.^52^ To further assess the level of agreement for the specific drugs assessed in this study, a third of the decisions included were cross-checked with NICE’s recommendations. A similar recommendation was found for 87% of these drug indications*.* As the evidence produced and emphasized by pharmaceutical developers is most likely the same across countries, the findings of this study could apply to many systems.

### Conclusions

The clinical and economic evidence used as a basis for reimbursement decisions for cancer drugs was found to be limited regarding the effects on patient-centered outcomes and characterized by major uncertainty and risk of bias. Given the resource limitations and need to ensure value for money in health care, the reimbursement of expensive treatments with low-quality evidence and unclear benefits is questionable. Decision makers should be cautious of the assumptions and evidence used in the economic evaluations of cancer drugs and consider how these uncertainties can be reduced.
