# Supplementary material for: Assessment of the clinical and cost-effectiveness evidence in the reimbursement decisions of new cancer drugs
Source: ESMO Open. 2022 Aug 28;7(5):100569. doi: 10.1016/j.esmoop.2022.100569 (PMC9588887; doi:10.1016/j.esmoop.2022.100569)
Supplement: Supplementary Material [file mmc1.docx]

**Assessment of the clinical and cost-effectiveness evidence in the reimbursement decisions of new cancer drugs**

**Supplementary Information**

Gabriella Chauca Strand,^a^ ,Carl Bonander^a^ ,Niklas Jakobsson^b^ ,Naimi Johansson^a,c^ , Mikael Svensson^a,d^

^a^ Health Economics and Policy, School of Public Health and Community Medicine, Institute of Medicine
University of Gothenburg, Medicinaregatan 18 A, PO Box 463, SE-405 30 Gothenburg

^b^ Karlstad Business School, Karlstad University. Faculty of Arts and Social Sciences, Karlstads Business School,

651 88 Karlstad, Sweden

^c^ University Health Care Research Center, Faculty of Medicine, and Health, Örebro University, SE-701 82 Örebro, Sweden

^d^ Department of Pharmaceutical Outcomes and Policy, University of Florida, Gainesville, Florida 32610, USA

**Table of contents**

[Supplementary Figure 1 (S1). Illustration Of Sample Selection 3](#_Toc111753301)

[Supplementary Table (S1). List Of Drugs 4](#_Toc111753302)

[Supplementary Table 2 (S2). Data Extraction Template 8](#_Toc111753303)

[Supplementary Table 2 (S3). Model Specifications 10](#_Toc111753304)

[Supplementary Table 4 (S4). The Final Decision And The Cost Per QALY 11](#_Toc111753305)

[Supplementary Table 5 (S5). Differences In QALY Gain By Type Of Evidence-Basis 12](#_Toc111753306)

[Supplementary Table 6 (S6). Results On Logistic Regressions With All Variables 13](#_Toc111753307)

# Supplementary Figure 1 (S1). Illustration of sample selection

Fig 1 Flowchart over sample selection based on TLV's list of applications over cancer drugs.


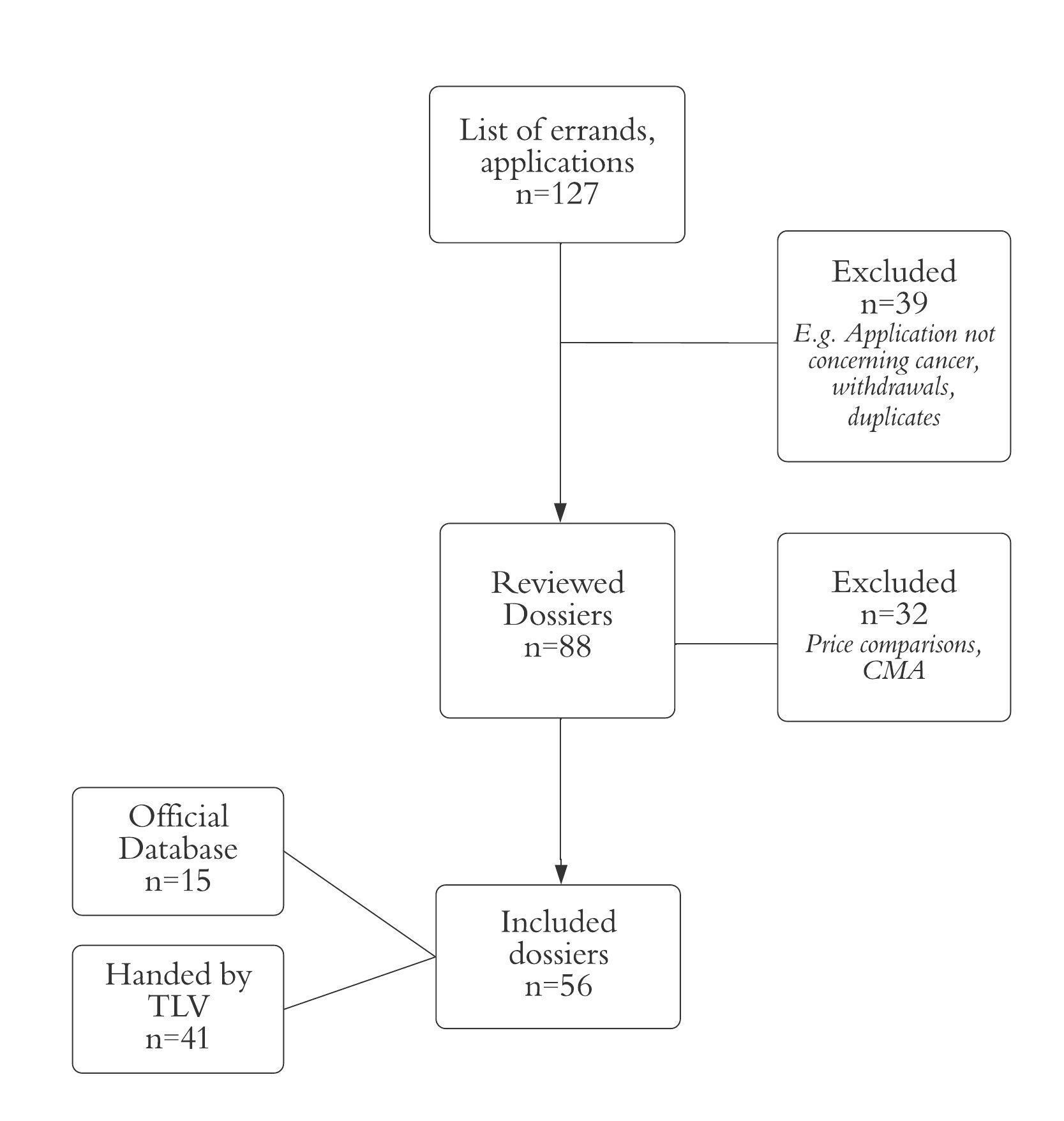


CMA, cost-minimization analysis. In a few selected dossiers (n=4), two appraisals for different specific drug indications were found. These appraisals were regarded independently.

# Supplementary Table (S1). List of drugs

Table 1 List of drugs included in the study

|  | |  |  | |  |
| --- | --- | --- | --- | --- | --- |
| Drug | Indication | | | Decision date | |
|  |  | |  | |  |
| Iressa | Locally advanced or metastatic NSCLC with EFGR-mutations | |  | | 2010-02-26 |
| Revlimid | Multiple myeloma with at least one prior course of treatment | |  | | 2010-06-30 |
|  |  | |  | |  |
| Afinitor® | Advanced RCC progressed under or after treatment with a VEGF-inhibitor | |  | | 2010-09-07 |
|  |  | |  | |  |
| Tasigna® | Newly diagnosed Ph+ CML | |  | | 2011-10-07 |
|  |  | |  | |  |
| Zelboraf | Non-resectable or metastatic melanoma with BRAF V600- mutation | |  | | 2012-10-25 |
|  |  | |  | |  |
| Inlyta® | Advanced RCC | |  | | 2013-01-31 |
| Xalkori® | Previously treated ALK+ advanced NSCLC | |  | | 2013-06-04 |
| Adcetris | Recurrent/refractory CD30+ Hodgkin’s Lymphoma and sALCL | |  | | 2013-06-20 |
| Bosulif® | Ph+ CML in chronic, accelerated or blast phase with previous treatments | |  | | 2013-10-03 |
|  |  | |  | |  |
| Xalkori® | Previously treated ALK+ advanced NSCLC | |  | | 2014-02-27 |
| Tafinlar | Non-resectable or metastatic melanoma with BRAF V600-mutation | |  | | 2014-02-20 |
| Erivedge | Advanced basal cell carcinoma | |  | | 2014-03-03 |
| Jakavi | Disease-related splenomegaly or symptomatic adults with primary myelofibrosis | |  | | 2014-04-04 |
|  |  | |  | |  |
| Xtandi | Metastatic castrate-resistant prostate cancer | |  | | 2014-05-27 |
| Stivarga | Metastatic colorectal cancer with previous treatments or ineligible for available treatments | |  | | 2014-06-03 |
|  |  | |  | |  |
| Imnovid | Recurrent, refractory multiple myeloma with two previous treatments and progressed disease | |  | | 2014-06-18 |
|  |  | |  | |  |
| Cometriq® | Thyroid cancer | |  | | 2014-11-20 |
|  |  | |  | |  |
| Tyverb | Metastatic, HR- breast cancer with previous treatments and progressed disease. | |  | | 2015-02-02 |
|  |  | |  | |  |
| Lynparza | Platinum-sensitive, recurrent epithelial ovarian, fallopian, or peritoneal cancer with BRCA-mutations | |  | | 2015-02-24 |
|  |  | |  | |  |
| Zydelig | Chronic lymphocytic leukemia. | |  | | 2015-02-24 |
| Zydelig | Follicular lymphoma refractory to two previous treatments | |  | | 2015-02-24 |
| Iclusig | Chronic myelogenous leukemia and Ph+ acute lymphocytic leukemia | |  | | 2015-03-27 |
|  |  | |  | |  |
| Vargatef | Locally advanced or metastatic NSCLC after first line of treatment. | |  | | 2015-05-21 |
| Zytiga | Metastatic castrate-resistant prostate cancer after failure of ADT and prior to chemotherapy treatment | |  | | 2015-05-21 |
| Zytiga | Metastatic castrate-resistant prostate cancer progressed during or after chemotherapy | |  | | 2015-05-21 |
| Imbruvica | Chronic lymphocytic leukemia | |  | | 2015-06-12 |
| Imbruvica | Refractory mantle-cell lymphoma | |  | | 2015-06-12 |
| Lenvima | Metastatic thyroid cancer, refractory to RAI | |  | | 2015-12-02 |
| Zykadia | ALK+ non-small cell lung cancer previously treated with Crizotinib | |  | | 2015-12-11 |
| Stivarga | Unresectable, metastatic GIST progressed on previous treatment or ineligible for available treatment | |  | | 2016-02-04 |
| Erivedge | Advanced basal cell carcinoma | |  | | 2016-04-27 |
| Xalkori® | ALK+ non-small cell lung cancer, first line treatment | |  | | 2016-06-16 |
| Mekinist | Unresectable, metastatic melanoma with BRAF V600 mutation | |  | | 2016-06-16 |
| Cotellic | Unresectable, metastatic melanoma | |  | | 2016-06-29 |
| Stivarga | Metastatic colorectal cancer with previous treatments or ineligible for treatment alternatives | |  | | 2016-09-07 |
| Faslodex® | Locally advanced or metastatic breast cancer in post-menopausal women | |  | | 2016-09-23 |
| Lonsurf | Metastatic colorectal cancer with previous treatment or ineligible for treatment alternatives | |  | | 2016-09-23 |
| Cabometyx® | Advanced renal cell carcinoma | |  | | 2017-04-20 |
| Ibrance | Locally advanced or metastatic HR+, HER2- breast cancer | |  | | 2017-06-15 |
| Tagrisso | Non-small cell lung cancer with T7900-mutation | |  | | 2017-09-28 |
| Darzalex | Recurrent, refractory multiple myeloma treated with previous therapies (monotherapy) | |  | | 2017-11-24 |
|  |  | |  | |  |
| Darzalex | Recurrent, refractory multiple myeloma treated with previous therapies (combination therapy) | |  | | 2017-11-24 |
|  |  | |  | |  |
| Alecensa | ALK+ non-small cell lung cancer previously treated with Crizotinib | |  | | 2017-11-24 |
| Faslodex® | ER+ locally advanced or metastatic breast cancer without previous endocrine treatment | |  | | 2018-01-25 |
|  |  | |  | |  |
| Kisqali | Locally advanced or metastatic HR+, HER- breast cancer as initial treatment | |  | | 2018-01-25 |
| Rydapt | Acute myeloid leukemia with FLT3-mutation | |  | | 2018-01-30 |
| Ibrance | Locally advanced or metastatic HR+, HER2- previously treated with endocrine treatment | |  | | 2018-02-22 |
| Cabometyx® | Advanced renal cell carcinoma | |  | | 2018-03-22 |
|  |  | |  | |  |
| Alecensa | First-line treatment for ALK+ non-small cell lung cancer | |  | | 2018-04-20 |
| Ninlaro | Multiple myeloma with at least one previous treatment | |  | | 2018-05-17 |
|  |  | |  | |  |
| Zytiga | High risk, metastatic hormone sensitive prostate cancer | |  | | 2018-06-14 |
| Mekinist | Melanoma, as adjuvant treatment | |  | | 2019-03-21 |
|  |  | |  | |  |
| Venclyxto | Chronic myeloid leukemia as second line treatment | |  | | 2019-04-26 |
| Xtandi | High-risk, non-metastatic prostate cancer | |  | | 2019-06-13 |
|  |  | |  | |  |
| Lorviqua | ALK+ non-small cell lung cancer | |  | | 2019-09-26 |
|  |  | |  | |  |
| Zejula | Maintenance treatment for relapsed ovarian cancer | |  | | 2019-11-22 |
| Lynparza | Ovarian, fallopian, or peritoneal cancer with BRCA-mutation | |  | | 2019-12-12 |
| Imbruvica | Recurrent or refractory mantle-cell lymphoma | |  | | 2020-03-19 |
| Venclyxto | Untreated chronic lymphocytic leukemia | |  | | 2020-08-27 |
| Vitrakvi | Solid tumors with NTRK-gene fusion | |  | | 2020-10-22 |
|  |  | |  | |  |

ADT; androgen deprivation therapy, ALK+; anaplastic lymphoma kinase-positive; CML; chronic myeloid leukemia, EGFR; epidermal growth factor receptor, ER+; estrogen receptor-positive, GIST; gastrointestinal stromal tumor, HR-; hormone-receptor negative; HR+; hormone-receptor-positive, HER2-; human epidermal growth factor receptor 2, NSCLC; non-small cell lung cancer, NTRK; neurotrophic tyrosine receptor kinase, Ph+; Philadelphia chromosome-positive, RAI; radioiodine therapy, RCC; renal cell carcinoma, sACLC; systemic anaplastic large cell lymphoma, VEGF; vascular endothelial growth factor

# Supplementary Table 2 (S2). Data extraction template

**Table 1** Template for data extraction

|  | **Category** | **Code** | **Focus, explanation** | **Example** |
| --- | --- | --- | --- | --- |
| **Application synopsis** | Application | Type of application | What does the application of the pharmaceutical regard? | New application, application for change in indication |
|  | | | | |
| **Disease Description** | Disease | Disease description | Focus is primarily on disease prevalence | Number of eligible patients, classified, not stated |
|  |  | Severity | What level of severity is the disease categorized as? Is there any explanation to it? | Highly severe, moderate severity – incurable, high mortality |
|  | | | | |
| **Pharmacologic Evidence** | Clinical efficacy | Primary outcome measures | Which outcomes are primarily looked at: surrogate measures or clinical outcomes? | HRQoL, overall survival, progression free survival, DFS |
|  |  | Statistical Significance of clinical outcomes | Is any statistically significant result found on clinical outcomes? |  |
|  |  | Study design | How is the study designed? Observational or experimental? | RCT, single-arm study, double-blinded RCT, not stated |
|  |  | Comparator | What kind of control is used? | Active control, placebo control |
|  | | | | |
| **Health Economics** | Economic Evaluation | Type of evaluation |  | CUA, CEA, CMA |
|  |  | Choice of comparator | What comparator is used in the economic evaluation? | Current treatment, no treatment |
|  |  | Underlying study | On what basis is the evidence for the evaluation retrieved? Are the treatments being compared directly or are indirect comparisons made? | RCTs, meta-analysis of RCTs, network-metaanalysis, single-arm trial |
|  |  | ICER | What is the result of the economic evaluation? Focusing only on the incremental cost-effectiveness ratio. | Cost per QALY, cost per life year gained |
|  | | | | |
| **Decision Summary** | Reimbursement decision | Decision | What is the stated decision regarding the drug? | Approval, restricted approval, rejection |
|  | Conditional Decision | Condition | What is the stated condition for approval? | Development of economic evidence |
|  | Decisive Criteria | Social Value | What factors are explicitly stated in the description of the final decision? | Level of severity, cost-effectiveness, uncertainty, lack of alternative treatments |

# Supplementary Table 2 (S3). Model specifications

| **Table 2** Logistic regression models | | | |  |  | | |
| --- | --- | --- | --- | --- | --- | --- | --- |
|  | |  |  | AIC | BIC | |  |
|  | |  |  |  |  | | |
| Model 1 | | Decision = F (Cost per QALY) | | 20.51 | 24.45 |  |  |
|  | |  |  |  |  |  |  |
| Model 3 | | Decision = F (cost per QALY, study design) | | 19.84 | 25.76 |  |  |
|  | |  |  |  |  |  |  |
| Model 4 | | Decision = F (cost per QALY, treatment alternative) | | 22.50 | 28.41 |  |  |
|  | |  |  |  |  |  |  |
| Model 5 | | Decision = F (cost per QALY, direct evidence on fin. outcome) | | 17.49 | 23.28 |  |  |
|  | |  |  |  |  |  |  |
| Model 6 | | Decision = F (cost per QALY, direct evidence on fin. outcome, treatment alternative) | | 19.48 | 27.22 |  |  |
|  | |  |  |  |  |  |  |
| Model 8 | | Decision = F (cost per QALY, type of comparison) | | 22.10 | 28.02 |  |  |
| Model 9 | | direct evidence on fin. outcome, treatment alternative | |  |  |  |  |
| Model 10 | | Decision = F (cost per QALY, direct evidence on fin. outcome, type of comparison) | | 19.23 | 26.96 |  |  |
| Model 11 | | Decision = F (cost per QALY, economic comparison, treatment alternative) | | 24.10 | 31.98 |  |  |
| All variables^[[1]](#footnote-1)^ | | Decision = F (cost per QALY, direct evidence on final outcomes, treatment alternative, type of comparator) | | 21.21 | 20.87 |  |  |
|  | | Decision = F (cost per QALY, study design, treatment alternative, type of comparator) | | 22.89 | 32.74 |  |  |
|  |  |  | | | | | |

# Supplementary Table 4 (S4). The final decision and the cost per QALY

| **Table 4** Cost per QALY over different decision outcomes (in Swedish krona, SEK1 = €0,0983) | | | | | | | | | |
| --- | --- | --- | --- | --- | --- | --- | --- | --- | --- |
|  | Decision | |  | All Decision Categories | | | | |  |
|  | Approval | Rejection |  | Approval | Conditional | Restricted | Restricted and conditional | Rejection |  |
| Mean cost per QALY | 749, 000 | 1, 380 000 |  | 682, 100 | 844, 800 | 774, 900 | 890, 700 | 1, 384200 |  |
| Median cost per QALY | 785, 000 | 1, 130, 000 |  | 755, 00 | 880, 000 | 815, 000 | 900, 000 | 1, 130 000 |  |
| Min cost per QALY | 275, 000 | 950,000 |  | 275, 000 | 710, 000 | 295, 000 | 822, 000 | 950, 000 |  |
| Max cost per QALY | 1 100, 000 | 2, 400 000 |  | 1,100 000 | 964, 000 | 1, 090 000 | 950, 000 | 2, 400 000 |  |
|  |  |  |  |  |  |  |  |  |  |
|  |  |  |  |  |  |  |  |  |  |

# Supplementary Table 5 (S5). Differences in QALY-gain by type of evidence-basis

| **Table 5** Differences in QALY-gain between types of evidence-bases, t-test | | | | | | | | | | | | | |
| --- | --- | --- | --- | --- | --- | --- | --- | --- | --- | --- | --- | --- | --- |
|  | | |  | Clinical Based | | | | 95% Confidence interval | T | | p-value | |  |
|  | | N | | | | Mean | SD |  | |  | |  | |
| Clinical Based | | 16 | | | 0.478 | | 0.068 | [0.33 – 0.62] | |  | |  | |
| Surrogate Based | | 15 | | | 0.995 | | 0.185 | [0.60 – 1.39] | |  | |  | |
| Difference | |  | | 0.516 | | | 0.192 | [0.12 – 0.91] | | 2.6855 | | 0.011 | |
|  |  |  | | | | | | | | | | | |

# Supplementary Table 6 (S6). Results on logistic regressions with all variables

| **Table 6** Logistic regressions on the likelihood of reimbursement. All variables | | | |
| --- | --- | --- | --- |
|  | (1) | (2) | |
|  |  |  |  |
| Cost per QALY | -0.100*** | -0.133** | |
|  | (0.035) | (0.042) | |
|  |  |  | |
| Direct evidence on final outcomes: Yes | -0.060  (0.073) |  | |
|  |  |  | |
| RCT |  | -0.230 | |
|  |  | (0.139) | |
| No treatment alt. | 0.010 | -0.069 | |
|  | (0.062) | (0.086) | |
| Direct comparison | -0.037 | 0.061 | |
|  | (0.072) | (0.084) | |
| Obs. | 51 | 53 | |
| Pseudo R^2^ | 0.75 | 0.73 | |
|  | | | |
| *** p<0.01, ** p<0.05, * p<0.1. Results in marginal effects. Standard errors are in parenthesis.  The cost per QALY is displayed in terms of 100 000 SEK increases. QALY, quality-adjusted life years; RCT, randomized controlled trial | | | |
|  | | | |

1. Due to multicollinearity between the variables study design and direct evidence on final outcomes, two separate regressions including all other variables were created. [↑](#footnote-ref-1)
